# From ion channel biology to clinical practice: suzetrigine as a precision analgesic

**DOI:** 10.3389/fmed.2026.1897421

**Published:** 2026-07-29

**Authors:** Amaya L. Bravo, Stacey I. Ajala, Michael F. Ayo, Brian J. Piper, Eric R. Wengert

**Affiliations:** 1 Geisinger College of Health Sciences, Scranton, PA, United States; 2 Department of Medical Education, Geisinger College of Health Sciences, Scranton, PA, United States; 3 Center for Pharmacy Innovation and Outcomes, Geisinger, Danville, PA, United States

**Keywords:** analgesia, Nav1.8, nociceptor, pain, peripheral nervous system, voltage-gated sodium channel, VX-548

## Abstract

Acute pain remains one of the most prevalent and burdensome clinical challenges requiring pharmacological intervention, yet opioids—with their well-documented risks of respiratory depression, addiction, and overdose—remain a cornerstone of moderate-to-severe pain management in the absence of efficacious alternatives. Suzetrigine (VX-548) is a novel preferential inhibitor of the voltage-gated sodium channel (VGSC) isoform Nav1.8, recently approved by the US Food and Drug Administration for the treatment of moderate-to-severe acute pain. Through specific inhibition of Nav1.8, which is expressed predominantly in nociceptive sensory neurons of the peripheral nervous system, suzetrigine offers a mechanistically targeted approach to reduce pain at the level of the primary afferent, bypassing the central nervous system circuitry involved in opioid-associated adverse effects. In this review, we examine both the scientific foundation and clinical utility of suzetrigine, integrating evidence from molecular and structural biology, electrophysiology, preclinical animal models, and Phase II and III randomized controlled clinical trials. We discuss the unique allosteric mechanism by which suzetrigine selectively inhibits Nav1.8 to suppress nociceptor excitability, and review six trials, five of which show evidence demonstrating significant analgesic efficacy relative to placebo, and a favorable safety profile. We discuss key limitations of the current clinical evidence base including modest, and sometimes nonexistent efficacy, albeit at the tested doses. Overall, suzetrigine may serve as a proof-of-concept for the development of other VGSC isoform-specific modulators as targeted therapeutic intervention for a range of diseases. Moreover, the development of cost-effective alternatives to prescription opioids may have substantial population health benefits. Further study with more varied pain types with longer durations including thoroughly examining sex differences with suzetrigine as a monotherapy (i.e., with no use of “rescue” medications) relative to prescription opioids or nonsteroidal anti-inflammatory drugs (NSAIDs) will provide valuable information that could widely benefit acute pain patients and form a scaffolding to bridge the gap between the well-established preclinical foundation and clinical research to enhance the quality of life of those currently living with chronic pain.

## 1 Introduction

Acute pain is among the most common and burdensome symptoms encountered for which pharmacological intervention is necessary. Despite substantial efforts to develop novel analgesics, opioids remain commonly utilized for the treatment of moderate-to-severe pain, highlighting the general lack of efficacious alternatives that circumvent the harms associated with opioid therapy. Suzetrigine (VX-548) is a first-in-class preferential inhibitor of the VGSC Nav1.8 to treat acute pain. As Nav1.8 is nearly exclusively expressed in the peripheral nervous system (PNS), suzetrigine represents a targeted approach to attenuate pain sensation at the level of the nociceptor. Although Nav1.8 expression itself is mostly restricted to the PNS, primary sensory neurons broadly express VGSC isoforms. Transcriptomic profiling of human dorsal root ganglion (DRG) has indicated detectable expression of numerous different VGSC isoforms, although functional contribution is not entirely clear (1). By bypassing central nervous system (CNS) neural circuitry involved in reward and respiration, suzetrigine seeks to offer pain reduction without the appreciable addiction potential and negative impacts on respiratory function associated with opioid-based pain management approaches and additionally provides substantial advantages relative to over-the-counter analgesics (NSAIDs, etc.). In this review, we discuss the scientific foundation for suzetrigine as a novel analgesic, the preclinical and clinical evidence for its use, and the overall impact that suzetrigine has in the context of pain management and the development of other precision medicine approaches in VGSC pharmacology.

## 2 Methods

A structured literature search was conducted to identify relevant peer-reviewed articles related to the basic mechanisms, pharmacological properties, preclinical results, and clinical impact of suzetrigine. Databases, such as PubMed Central and Google Scholar, were utilized to retrieve the articles referenced. During the search, keywords included but not limited to: *suzetrigine*, *Nav1.8 inhibitor*, *VX-548*, *opioid pain relief mechanisms*, *pain*, *pain relief*, *analgesic medications*, *nociceptive pain*, *suzetrigine clinical trials*. As of February 27, 2026, a search of “Suzetrigine OR VX-548” in PubMed yielded 91 results. After an initial screen of topical relevance, we included only articles that were peer-reviewed primary research articles in the English language that were (1) studies investigating the mechanisms of action and properties of suzetrigine or VX-548, (2) preclinical studies involving Nav1.8 inhibition in various model systems, (3) clinical studies including Phase II or III clinical trial data on suzetrigine, or in a few limited cases (4) other review articles/commentaries focusing on suzetrigine efficacy, cost (2), adverse effects or VGSC isoform-specific pharmacology. In a few places, we summarized the results of the clinical trials using Cohen’s *d* in which the magnitude of the effect size for group differences was expressed in terms of Cohen’s *d* where 0.2, 0.5, and 0.8 are small, medium, and large, respectively (3).

## 3 Background: scientific premise

VGSCs are central determinants of cellular excitability—they directly contribute to the generation and propagation of action potentials (APs) (4, 5). Upon membrane depolarization, voltage-gated sodium channels open and permit an influx of sodium ions into the cell. The incoming sodium initiates a feed-forward cycle of depolarization which is responsible for the upstroke of the AP. Within milliseconds of opening, voltage-gated sodium channels inactivate, and together with the opening of delayed-rectifier voltage-gated potassium channels, the membrane rapidly repolarizes comprising the downstroke of the AP (4, 5). Once at relatively negative membrane potentials, inactivated sodium channels recover and again become sensitive to open in response to depolarized membrane potentials. This cyclic progression from closed to open to inactivated states, forms the basis for each AP.

There are nine unique VGSC alpha subunits encoded by different genes: *SCN1A*, *SCN2A*, *SCN3A*, *SCN4A*, *SCN5A*, *SCN8A*, *SCN9A*, *SCN10A*, and *SCN11A* (6). Each of these genes is uniquely expressed in terms of cell type, tissue, and developmental timing of expression (Table 1) (6, 7). Based on their unique expression profile, it is unsurprising then that genetic variants in each of these genes can lead to highly specific syndromes/disorders (Table 1).

**Table 1:** Functional diversity of voltage-gated sodium channel alpha subunits.

Gene	Channel isoform	Primary cellular populations	Primary tissue	Developmental timing	Associated syndromes
*SCN1A*	Nav1.1	CNS neurons (inhibitory)	Brain, spinal cord	Low prenatally, increases postnatally	Epilepsy, autism spectrum disorders, Migraine, intellectual disability
*SCN2A*	Nav1.2	CNS neurons (excitatory)	Brain, spinal cord	Fetal development through adulthood	Epilepsy, ASD, ID
*SCN3A*	Nav1.3	CNS and PNS neurons	Brain, spinal cord	Expression peaks in prenatal and early neonatal periods	Epilepsy, congenital cortical malformation, ASD
*SCN4A*	Nav1.4	Skeletal muscle fibers	Skeletal muscle	Fetal development through adulthood	Hyperkalemic periodic paralysis, hypokalemic periodic paralysis, paramyotonia congenita, congenital myopathy
*SCN5A*	Nav1.5	Cardiomyocytes	Heart (atria, ventricles, conduction system)	Fetal development through adulthood	Long QT syndrome, Brugada syndrome, cardiac conduction disease, atrial fibrillation, dilated cardiomyopathy
*SCN8A*	Nav1.6	CNS neurons	Brain, spinal cord	Low prenatally - >, increases postnatally	Epilepsy, ASD, ID
*SCN9A*	Nav1.7	Nociceptive sensory neurons, sympathetic neurons	Dorsal root ganglia, trigeminal ganglia, autonomic ganglia	Fetal development through adulthood	Congenital insensitivity to pain, primary erythromelalgia, paroxysmal extreme pain disorder
*SCN10A*	Nav1.8	Nociceptive sensory neurons (C-fibers)	Dorsal root ganglia, peripheral nerves	Appears postnatally, persists into adulthood	Small fiber neuropathy, erythromelalgia
*SCN11A*	Nav1.9	Nociceptive sensory neurons	Dorsal root ganglia, enteric neurons	Postnatal and adult expression	Familial episodic pain syndrome; congenital insensitivity to pain; painful neuropathy

Voltage-gated sodium channels are the molecular targets of many compounds including both environmental toxins and therapeutic drugs (7, 8). Drugs that inhibit voltage-gated sodium channels are used to treat cardiac arrhythmias, severe epilepsy, neuropathic pain, and as local anesthesia (8–11). Yet, because most currently available drugs (i.e., oxcarbazepine, lamotrigine, etc.) are relatively nonselective for one channel isoform relative to others, pharmacological agents targeting voltage-gated sodium channels have substantial adverse effects (i.e., dizziness, drowsiness, headache, cardiac arrhythmia, etc.) (10).

Fueled by a greater structural understanding of each of the eukaryotic voltage-gated sodium channels (12–14), the past few years has brought about clear gains in developing VGSC isoform-specific small-molecule inhibitors to treat epilepsy and chronic pain (12, 15–17). Preferential inhibitors are currently in development for Nav1.2 and Nav1.6 for the treatment of severe epilepsy (16, 18), and Nav1.7 and Nav1.8 for the treatment of pain (12, 15, 19–21). The scientific premise is straightforward: through greater preferential effect on particular VGSC isoforms expressed in certain cellular populations and/or tissues, clinicians will be able to provide more targeted and efficacious treatment, meanwhile limiting the adverse effects through inhibition of other channels beyond the intended target (i.e., blocking Nav1.5 in the heart when the target is Nav1.8 in the PNS).

Although nociceptors express multiple VGSC isoforms (1), there are numerous scientific reasons that selective inhibition of Nav1.8 should result in meaningful analgesia with advantages over non-selective blockers. For one, co-expression of numerous VGSC isoforms does not necessarily imply functional redundancy. Nav1.8, due to its relatively depolarized activation, uniquely contributes to repetitive AP generation in nociceptors (22). Further, the advantage of isoform selectivity relative to non-selective VGSC blockers is derived primarily due to therapeutic index rather than peak efficacy: because local anesthetics inhibit multiple isoforms, they cannot be delivered systemically without dose-limiting toxicity, and therefore are largely confined to local administration for treating pain. Sparing these off-target isoforms from being inhibited is what enables Nav1.8-selective inhibition to provide systemic, orally bioavailable analgesia with an acceptable safety profile.

## 4 Preclinical studies

**Figure 1:**
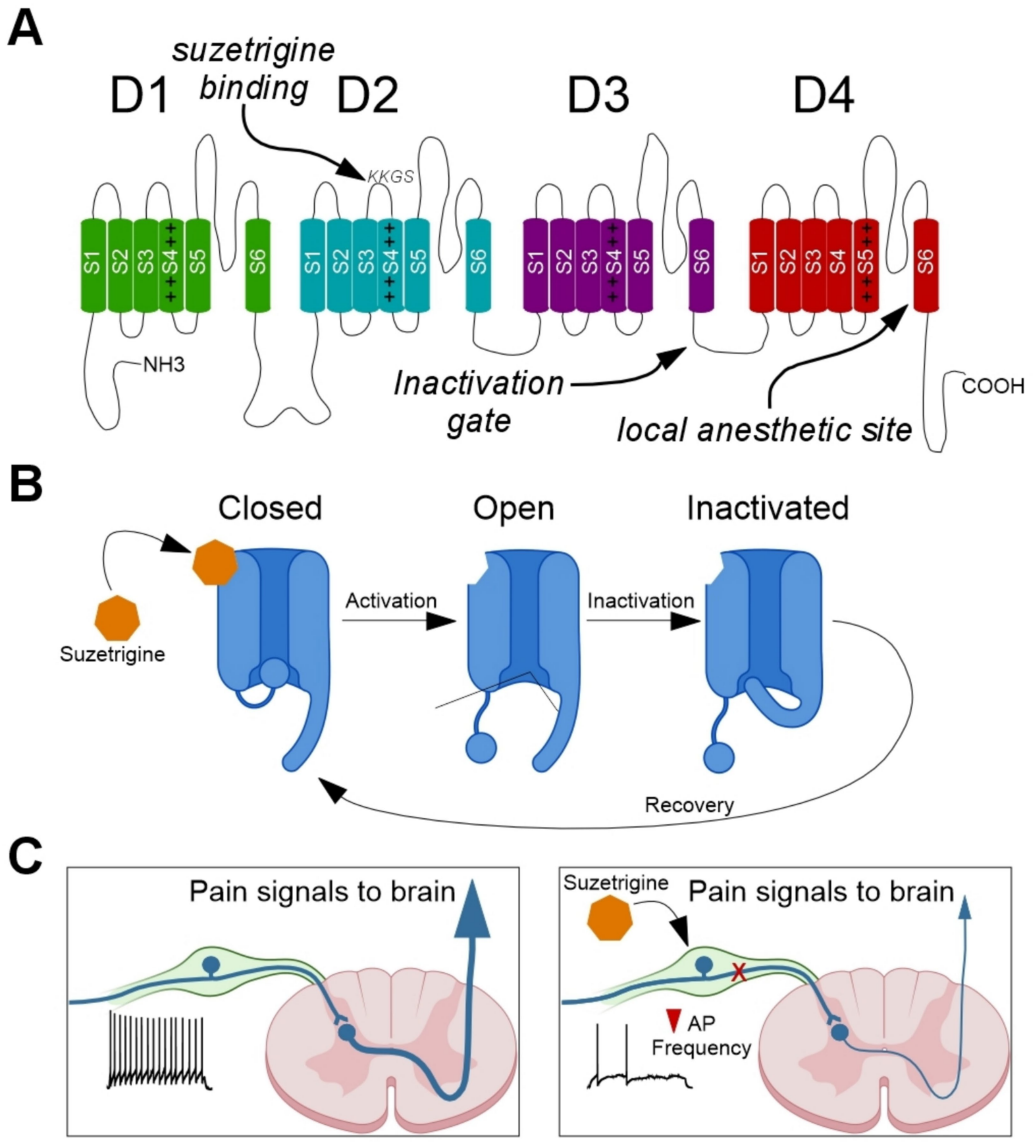
Mechanism of action of suzetrigine. **(A)** Primary sequence of voltage-gated sodium channel Nav1.8. Domains I through IV, each with six transmembrane segments (S1-6) are shown in unique colors. Suzetrigine binds a unique motif (KKGS) in the extracellular loop between the S3 and S4 of DII. This site is notably distinct from the local anesthetic site in S6 of DIV, and is also distinct from regions of the channel most associated with fast inactivation (the loop DIII and DIV). **(B)** Cartoon depiction suzetrigine preferential binding to voltage-gated sodium channels in the closed state. This binding preference is unique relative to other voltage-gated sodium channel blockers which exhibit enhanced affinity for depolarized channel states (open and inactivated). **(C)** Due to high levels of Nav1.8 expression in nociceptive neurons in the dorsal root ganglion, suzetrigine reduces the frequency of action potentials (APs) generated in response to painful stimuli. The reduction in APs in this first-order neuron, results in dampened pain signal being transmitted up the spinal cord to the brain. Ultimately this results in an attenuation of pain perception.

Suzetrigine was developed as a small-molecule specific inhibitor of Nav1.8 to treat pain (19, 23, 24). Because Nav1.8 is expressed exclusively in the DRG of the PNS where it primarily contributes to the cellular excitability of nociceptive afferents, selective blockade of Nav1.8 would be expected to reduce pain sensation through direct attenuation of the first-order nociceptor neuron (25) (Figure 1). This mechanism of action contrasts with that of opioid analgesics which heavily influence CNS function to attenuate the perception of pain (26), and NSAID-based analgesia which is exerted through reducing peripheral inflammation and influencing brain regions involved in descending pain modulation (27).

Preclinical studies have provided the foundation for suzetrigine as a potent and selective inhibitor of Nav1.8. Osteen et al. (19) showed blockade of TTX-resistant (presumably mediated by Nav1.8) in cultured primary human DRG neurons by VX-548 with an IC50 of 0.68 +/− 0.16 nM (19). This study also examined the impact of suzetrigine on each of the VGSC isoforms and showcased more than 31,000-fold greater selectivity for Nav1.8 than for any of the other channel isoforms (19). This report further demonstrated that suzetrigine inhibited Nav1.8 via a unique allosteric mechanism relative to other VGSC modulators that typically bind at the local anesthetic site (28, 29). Domain swapping experiments identified a particularly important role for a sequence of amino acids: lysine, lysine, glycine, and serine (KKGS), found in the S3 to S4 extracellular loop of the second voltage-sensing domain (VSD2) of Nav1.8, but not other isoforms (19) (Figure 1). Further supporting this unique allosteric mechanism is evidence that co-application of suzetrigine with bupivacaine or ropivacaine, traditional VGSC blockers that bind at the local anesthetic site resulted in simple additive inhibition of Nav1.8 currents, with no evidence of antagonism or competition (19).

Electrophysiological experiments also identified that the Nav1.8 inhibitor suzetrigine exhibits the unusual property in that its inhibitory effect is dramatically reduced by depolarization (19, 21). These results imply that suzetrigine most strongly binds to the resting state (i.e., closed) voltage-gated channel, and that opening of the channel alters the binding site in the extracellular cleft formed by the S3 and S4 helices to reduce the affinity of the drug by more than 100 times (21, 30).

Through targeted inhibition of Nav1.8, theoretically, suzetrigine should have profound effects on the excitability of DRG neurons to reduce the frequency of AP generation and potentially influence passive membrane and AP properties. Supporting this view, suzetrigine exhibited a dose-dependent impact on the probability of AP generation in an isolated human DRG neuron (19, 31). In terms of individual AP properties, suzetrigine decreased the peak of the AP without a strong effect on the rising phase or the AP threshold voltage (20, 31). Suzetrigine application also led to a strong narrowing of the AP (20). These results suggest that Nav1.7, which is more sensitive to activation at more hyperpolarized voltages, contributes strongly to the upstroke of the AP in DRG neurons while Nav1.8 with its slower inactivation kinetics and resurgent sodium currents (32, 33), influences the falling phase of the AP more strongly (20). In measurements of repetitive neuronal spiking, suzetrigine application typically reduced the frequency of repetitive AP generation (19, 20, 31). Overall, these experiments provide the physiological foundation for the overall scientific mechanism that suzetrigine is an effective and potent inhibitor of Nav1.8 and nociceptive afferent excitability.

There are also a few lines of evidence from *in vivo* rodent studies that support the case that suzetrigine is an effective analgesic and that its use is well tolerated with reduced risk of addiction or dependency. Mice were examined to identify the effect of suzetrigine in numerous distinct pain models including the formalin test, complete Freund’s adjuvant (CFA) induced thermal hypersensitivity test, and mechanical hyperalgesia in the partial sciatic nerve injury-induced neuropathic pain model (34). Suzetrigine treatment at 1 mg/kg and 10 mg/kg reduced nociceptive behavior when administered 30 min before intraplantar formalin injection (34). Similarly, suzetrigine produced time and dose-dependent analgesia in response to CFA-induced thermal hypersensitivity (34). In the sciatic nerve injury model of neuropathic pain, suzetrigine (10 mg/kg) rapidly increased mechanical withdrawal thresholds within 30 min, but by 60 min the analgesic effect was no longer observed (34). Successive daily treatment with suzetrigine did not influence the magnitude of analgesia suggesting that repetitive dosing over this time scale did not induce strong tolerance or altered physiological response to suzetrigine (34). As evidence for the safety of suzetrigine, abrupt cessation of chronic treatment (after 30 days) in rats did not impact body weight or body temperature, two measures in which morphine-treated rats showed significant differences following withdrawal (19). Spontaneous motor function in the 14 h following abrupt cessation of treatment was also not different in suzetrigine-treated mice relative to vehicle controls, while morphine-treated rats displayed less movement (19). Notwithstanding that there are a limited number of reports of behavioral studies investigating suzetrigine directly (19, 34), the available peer-reviewed evidence is supportive of the view that suzetrigine is safe and effective at ameliorating pain in rodent models.

Notably, these *in vivo* results must be considered in the context of a substantial difference in potency depending on species. Suzetrigine inhibited Nav1.8-mediated currents in mouse DRG neurons with an IC50 of ~0.35 μM while the IC50 for human Nav1.8 was ~0.68 nM (19, 34). The difference in potency by species taken alongside the findings that suzetrigine did exhibit analgesic effects in rodents emphasizes the selectivity of suzetrigine and provides some explanation as to why rodent efficacy data were not entirely relied upon to inform human dose selection, which instead drew on human Nav1.8-based models.

## 5 Pharmacokinetic considerations

The absorption, distribution, metabolism, and elimination of suzetrigine have been characterized in adults (19) and in the package insert.^1^ Based on the package insert, the time to maximum concentration following fasted-state oral administration is 3.0 h, with 99% protein binding. Suzetrigine is metabolized via hepatic cytochrome P450, particularly 3A4 to its biologically active metabolite M6-suzetrigine (35). The elimination half-life is about one-day (i.e., 23.6 h for suzetrigine and 33.0 h for M6-suzetrigine). The excretion is about half (49.9%) in feces with almost all of the remainder (44.0%) in urine. Of note, suzetrigine exhibits substantial tissue distribution including the CNS indicating that its favorable central safety profile is driven by peripheral localization of its molecular target and not by restricted brain penetration (see text footnote 1).

A single rat study found evidence for pronounced sex differences in suzetrigine metabolism including a twelve-fold greater maximum concentration, double the half-life and a fifty-fold increased area under the concentration curve in females following oral administration (35). These robust sex differences were replicated with doubling of the half-life and a six-fold greater area under the concentration curve in females subsequent to intraveneous administration. These findings, and their functional implications, should be investigated more closely in other animal models and humans including in trials to determine if males require higher doses to reach similar plasma levels and overcome potential sex-based differences in bioavailability. Structural modifications of suzetrigine with the development of a female and male specific formulation could potentially be necessary to achieve more equivalent pharmacokinetics in both sexes (24).

Considering this metabolic mechanism, suzetrigine would be expected to exhibit drug–drug interactions with other drugs that induce or inhibit CYP3A function and therefore should be used cautiously (24). Persons of reproductive age identifying as “women” should be cautioned to also consider the use of non-hormonal contraception suzetrigine, as a CYP3A substrate could lower plasma concentrations of progestin-based birth control. Pharmacists and other health care providers should also counsel patients to avoid use of grapefruit containing products (24).

Taken together, suzetrigine’s selective inhibition of the peripherally-expressed Nav1.8 positions it as a mechanistically-distinct analgesic that should make it a potential candidate for co-administration with opioids and NSAIDs in certain cases of acute moderate to severe pain. The mechanistic complementarity suggests that combining suzetrigine and opioids could achieve equivalent or superior analgesia with lower opioid doses- an attractive feature to reduce exposure to opioid-related adverse effects. Importantly however, co-administration of suzetrigine alongside other analgesic agents has yet to be investigated in animal models and clinical trials, although limited *in vitro* evidence suggests that targeting of voltage-gated sodium channels with traditional local anesthetic agents is mechanistically additive to sodium current inhibition provided by suzetrigine (19). Further clinical research regarding drug–drug interactions in the context of multimodal analgesia among varied patient populations is a pressing need.

## 6 Clinical trials

Suzetrigine has also recently been investigated in numerous randomized, double-blind clinical trials to provide direct evidence for safety, and efficacy in human patients experiencing acute pain. Of note, at the time of writing, the results of only five of these clinical trials have been published in peer-reviewed journals (36–38), while two Phase II and III clinical trials for painful diabetic peripheral neuropathy (NCT05660538) and lumbosacral radiculopathy (NCT06176196) are underway. Note that two of these publications each contain two trials (36, 37). A summary of the clinical trial findings is provided in Table 2.

**Table 2:** Summary of clinical trial information for suzetrigine.

Feature	Phase II RCT (37)	Phase III RCTs (36)	Phase III single-arm open-label (38)
Study design	Two phase 2, randomized, double blind, placebo and active controlled trials in acute postoperative pain after abdominoplasty or bunionectomy.	Two phase 3, randomized, double blind, placebo and hydrocodone bitartrate/acetaminophen controlled trials after abdominoplasty or bunionectomy.	Phase 3, single arm, open label study in surgical and non surgical acute pain.
Total enrollment/N	Abdominoplasty *N* = 303; bunionectomy *N* = 274.	Abdominoplasty *N* = 1,118; bunionectomy *N* = 1,073.	*N* = 256 received at least one dose of suzetrigine.
Sex breakdown	Predominantly women. Abdominoplasty female counts by arm: 75/76, 74/74, 73/76, 76/77; bunionectomy female counts by arm: 53/60, 57/62, 25/33, 50/60, 49/59.	Abdominoplasty: 1,098/1,118 (98%) female; bunionectomy: 912/1,073 (85%) female.	173/256 (67.6%) female; 83/256 (32.4%) male.
Pain model	Established acute postoperative pain models: abdominoplasty (soft tissue pain) and bunionectomy (bone pain).	Established acute postoperative pain models: abdominoplasty (soft tissue pain) and bunionectomy (bone pain).	Broad acute pain population: 222 surgical (86.7%) and 34 non surgical (13.3%).
Sample type	Adults 18 to 75 years with postoperative pain rated moderate or severe on VRS and at least 4 on NPRS.	Adults with postoperative pain rated moderate to severe on VRS and at least 4 on NPRS after surgery.	Adults 18 to 80 years with moderate or severe acute pain on VRS and at least 4 on NPRS after surgery or with new non surgical pain.
Dosing regimen	Abdominoplasty: high dose, 100 mg loading then 50 mg q12h; middle dose, 60 mg loading then 30 mg q12h; HB/APAP 5/325 mg q6h; placebo q6h. Bunionectomy: same high and middle dose arms plus low dose, 20 mg loading then 10 mg q12h; HB/APAP 5/325 mg q6h; placebo q6h.	Suzetrigine 100 mg loading dose then 50 mg q12h; hydrocodone bitartrate/acetaminophen 5/325 mg q6h; placebo for 48 h.	Suzetrigine 100 mg first dose then 50 mg q12h for 14 days or until pain resolution; rescue acetaminophen 650 mg and ibuprofen 400 mg q6h allowed.
Treatment duration	48 h.	48 h.	Up to 14 days or until pain resolution.
Primary efficacy vs. placebo	High dose VX-548 reduced pain vs. placebo: LS mean SPID48 difference 37.8 (95% CI 9.2 to 66.4) after abdominoplasty and 36.8 (95% CI 4.6 to 69.0) after bunionectomy; lower doses were similar to placebo.	Primary endpoint achieved in both trials: LS mean SPID48 difference 48.4 (95% CI 33.6 to 63.1; *p* < 0.0001) after abdominoplasty and 29.3 (95% CI 14.0 to 44.6; *p* = 0.0002) after bunionectomy.	No placebo comparator. At end of treatment, 83.2% (213/256) rated suzetrigine as good, very good, or excellent on patient global assessment.
Efficacy vs. opioid comparator	Active comparator included, but the main analysis compared each VX-548 dose with placebo; no direct efficacy conclusion versus HB/APAP was drawn.	Not superior to HB/APAP on SPID48: abdominoplasty difference 6.6 (95% CI -5.4 to 18.7; *p* = 0.2781); bunionectomy difference −20.2 (95% CI -32.7 to −7.7; *p* = 0.0016).	No active comparator arm.
Onset of meaningful relief	Not formally analyzed.	Median time to at least 2 point NPRS reduction: 119 min vs. 480 min placebo after abdominoplasty and 240 min vs. 480 min placebo after bunionectomy.	Not directly assessed in this single arm study.
Common adverse events	Headache and constipation were common with VX-548; the most common events occurring in at least 10% of any group included nausea, headache, constipation, dizziness, and vomiting in abdominoplasty, and nausea and headache in bunionectomy.	Most common adverse events occurring in at least 4% of any group were nausea, constipation, headache, dizziness, hypotension, and vomiting.	Headache (7.0%), constipation (3.5%), nausea (3.1%), fall (2.3%), and rash (2.0%).
Serious adverse events	Serious adverse events were rare; none were considered related to active treatment or placebo.	After abdominoplasty, serious adverse events were 2.5% with suzetrigine, 1.6% with HB/APAP, and 2.3% with placebo, and none were considered related; after bunionectomy, there were no serious adverse events.	2/256 (0.8%) serious adverse events, cellulitis and suicidal ideation; both were considered not related to suzetrigine.
Respiratory depression	Not reported	Not reported	Not reported
Euphoria/abuse signal	Not specifically reported as a trial endpoint in this paper.	Peripheral nonopioid mechanism is emphasized, but formal abuse signal testing was not presented as a trial endpoint in this report.	The paper states there is no evidence of addictive potential based on mechanism, preclinical data, and prior clinical adverse event data, but this was not a formal endpoint in this study.
CNS effects	Peripheral NaV1.8 selectivity is emphasized, but no formal CNS effects endpoint was reported.	Nav1.8 is described as not being expressed in the human brain or spinal cord, supporting lack of central opioid-like effects, but CNS effects were not a formal endpoint.	The paper states suzetrigine does not have CNS side effects associated with opioids based on high Nav1.8 selectivity, but this is mechanistic rationale rather than a dedicated endpoint.
Key limitation	Short treatment window (48 h) and no direct powered comparison versus Hydrocodone bitartrate (HB)/ acetaminophen (APAP).	Short duration (48 h) and postoperative acute pain models only.	Single arm design, no data beyond 14 days.

A Phase II study of suzetrigine in painful lumbosacral radiculopathy (NCT06176196), results of which were released only as a press release, revealed that suzetrigine produced a mean −2.02 point reduction from baseline on the 11-point Numeric Pain Rating Scale (NPRS) at Week 12, compared to −1.98 for placebo (58). Although the study was not powered for between-group statistical comparison, the near-identical within-group changes suggest no clinically meaningful incremental benefit of suzetrigine over placebo for this indication—a finding consistent with the high placebo response rates commonly observed in neuropathic pain trials (39).

Two phase-two randomized, double-blind trials were completed to evaluate suzetrigine in acute pain patients following abdominoplasty (NCT05034952) or bunionectomy (NCT04977336) (37). Patients were randomly assigned to high-dose suzetrigine (100 mg followed by 50 mg every 12 h), mid-dose suzetrigine (60 mg followed by 30 mg every 12 h), low-dose suzetrigine (20 mg followed by 10 mg every 12 h), hydrocodone bitartrate/acetaminophen (5 mg/325 mg every 6 h), or placebo, and were assessed for time-weighted sum of pain intensity difference over 48 h. Patients in the high-dose suzetrigine treatment group, but not the mid or low dose groups, experienced a significant reduction of pain relative to the placebo group for both abdominoplasty and bunionectomy within 48 h (37). Suzetrigine was therefore interpreted by the authors as comparable to pain management with hydrocodone/acetaminophen. Fewer patients withdrew from the high-dose suzetrigine group for lack of efficacy than from the hydrocodone/acetaminophen or placebo groups (37). Most adverse effects in the trials were mild or moderate, most commonly nausea, headache, constipation, dizziness, and vomiting. Of the few serious adverse events that occurred, none were considered by the site investigators to be directly related to suzetrigine treatment (37). Overall, these results suggested safety and possible efficacy of suzetrigine in the context of acute pain.

However, there have been numerous criticisms of the Jones et al. trial (2, 40, 41). First, females accounted for the vast preponderance (98.3%) of the abdominoplasty trial but disparities due to sex were less pronounced in the in the bunionectomy (55.5% female) trial. Second, at the time of writing the individual responsible for the Statistical Analysis Plan (SAP) had their information redacted (59–61). Third, the potential confounding role of NSAIDs across treatment groups appears to have not been fully taken into account. NSAIDs have a well-established role as opioid-sparing in perioperative procedures (42). A randomized controlled trial of ambulatory surgery patients determined that ibuprofen (1,200 mg/day) decreased pain at 72 h and decreased hydrocodone (5 mg) / acetaminophen (500 mg) usage at 48 (effect size = ~0.8) and 72 h (effect size = ~0.5) significantly more than placebo (43). As has been also pointed by others (41), ibuprofen with doses up to 1,600 mg/day was an allowable rescue medication in the Jones et al. study. Although the SAP indicated that the percent of participants using rescue medication, and the total rescue medication use from 0 to 48 h would be reported, both trials did not report on ibuprofen use making it challenging to untangle the effect of suzetrigine relative to NSAIDs (37). Fourth, the results from the positive control condition are difficult to interpret given that pain ratings over 48 h were not statistically different between hydrocodone 5 mg/acetaminophen 325 mg (every 6 h) and placebo in either the abdominoplasty or bunionectomy trials (Supplementary Figure 1 and Supplementary Table 1). This is surprising because hydrocodone/acetaminophen is among the most prescribed and distributed analgesics in the United States (44, 45). Jones et al. had a reasonable basis for selecting this combination as a positive control for comparison, as it was presumably expected to produce a clear, detectable analgesic signal—making the null finding against placebo particularly difficult to explain (37). Fifth, section 6.1.2 of the SAP noted that “Time to onset of “confirmed perceptible pain relief” and “meaningful pain relief” after the first dose of study drug” would be dependent measures (Vertex, 2022)^2^ but this was not reported (37). Based on our calculations, the Cohen’s *d* effect size at 48 h for the Sum of the Pain Intensity Differences with the high-dose VX-548 was 0.42 in the abdominoplasty trial and 0.41 in the bunionectomy trials. Although a large placebo response and lack of validated biomarkers make studying acute pain challenging (39), there are reasonable concerns as to whether these outcomes should be considered clinically meaningful (41) (Supplementary Table 1). Lastly, as the Patient Global Assessment (PGA) was part of the SAP but not the published report, this raises the potential concern of selective reporting (40).

In two additional randomized, double-blind, placebo- and active-controlled Phase III bunionectomy (NCT05553366) and abdominoplasty (NCT05558410) trials (36), adults with moderate-to-severe postoperative pain received a 100 mg oral loading dose followed by 50 mg every 12 h and were compared to both placebo and hydrocodone/acetaminophen over a 48-h treatment period (36). The N per group completing the trials was 168 to 396 in the abdominoplasty trial and 177 to 389 in the bunionectomy trial. The trials’ primary outcome was the time-weighted summed pain intensity difference over 48 h (SPID48), which showed that suzetrigine significantly reduced pain in both surgery models when compared to a placebo although significantly greater analgesia relative to hydrocodone/acetaminophen was not observed (36). Suzetrigine led to a faster onset of clinically meaningful analgesia than placebo, defined as a reduction of at least 2 points on the 0–10 NPRS. Median times to achieve this reduction were 119 min verses 480 min after bunionectomy (95% CI, 14.0 to 44.6; *p* = 0.0002) and 240 min versus 480 min after bunionectomy (95% CI, 14.0 to 44.6; *p* = 0.0002). Hydrocodone/acetaminophen produced more rapid initial pain relief than suzetrigine in the abdominoplasty trial. Interestingly, the incidence of vomiting/nausea, a common side-effect observed in response to opioid-based treatments, was lower in the suzetrigine-treated group relative to the hydrocodone/acetaminophen group in both studies as an important secondary outcome (36). Overall, these investigations suggest that suzetrigine was safe and well-tolerated.

**Figure 2:**
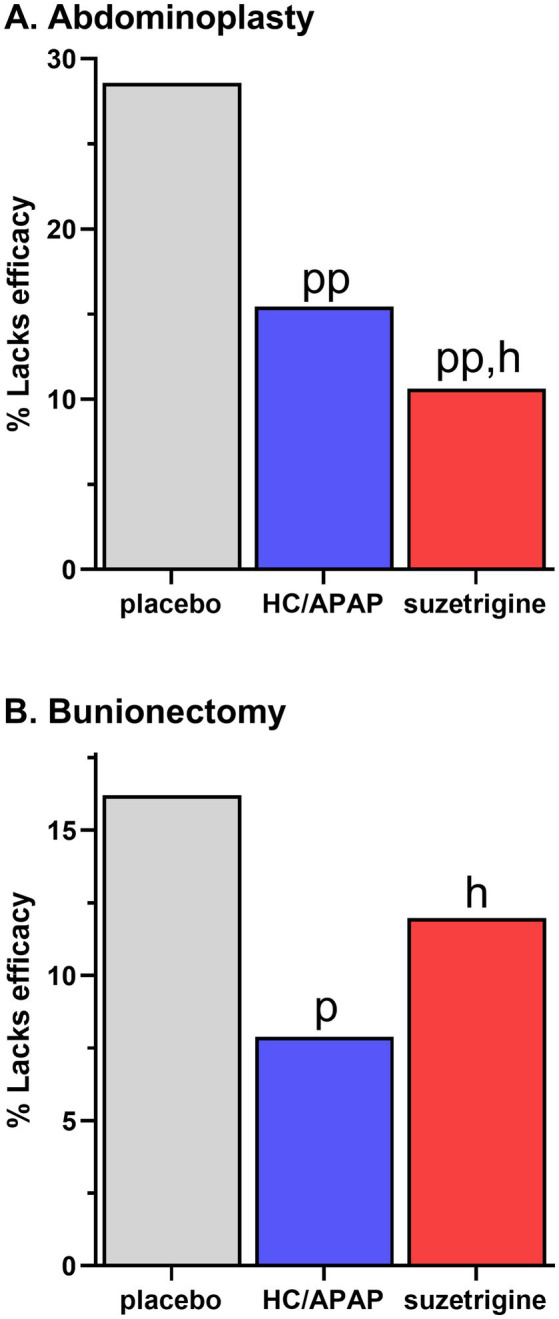
Percent of randomized and dosed participants who discontinued treatment due to lack of efficacy that received placebo, hydrocodone (5 mg) / acetaminophen (325 mg every 6 h, HC/APAP), or suzetrigine (100 mg loading, 50 mg every 12 h) in the abdominoplasty **(A)** or bunionectomy **(B)** trials of (36). Chi-square ^p^*p* < 0.05 or ^pp^*p* < 0.0005 versus placebo; ^h^*p* < 0.05 versus hydrocodone / acetaminophen.

The percent of randomized and dosed participants who discontinued treatment due to lack of efficacy was not determined or analyzed in (36). In the abdominoplasty trial, both active treatments reduced efficacy-related dropout relative to placebo (28.6%), with suzetrigine (10.6%) outperforming hydrocodone/acetaminophen (15.5%; *p* < 0.05) (Figure 2A). Interestingly, the bunionectomy trial told a contrasting story: suzetrigine’s dropout rate due to lack of efficacy (12.0%) was not significantly different from placebo (16.2%), whereas hydrocodone/acetaminophen (7.9%) was (Figure 2B). This failure to separate from placebo on a real-world retention measure in the bunionectomy trial reinforces concerns about suzetrigine’s analgesic adequacy in that surgical context. As in the Jones et al., trial, there are also concerns about the clinical meaning underlying the analgesic effect observed in the Bertoch et al., study. The Cohen’s *d* for the SPID at 48 h in the abdominoplasty trial was moderate at 0.531 imputed and 0.505 as treated, but the effect size was only small in bunionectomy trial at 0.319 imputed and 0.296 as treated. Second, the sample characteristics were analyzed by the Institute for Clinical and Economic Review (ICER) and the diversity by sex in both trials (85–98% female) was rated “Poor”.^3^

Suzetrigine’s efficacy and safety across larger acute pain groups has been reinforced by Phase III open-label safety research that assessed the medication in people with moderate-to-severe acute pain of both surgical and non-surgical origin for up to 14 days (38). In 256 individuals with moderate or severe pain, at least one dose of suzetrigine was delivered (100 mg initial dose followed by 50 mg every 12 h) before assessing safety and tolerability as well as patient perceptions of effectiveness (38). Suzetrigine treatment was generally well-tolerated with only mild or moderate adverse effects observed including headache, nausea, constipation, rash, and others (38). Two (0.8%) patients in the study had serious adverse events: One patient experienced suicidal ideation, and one experienced cellulitis. Overall, patients perceived pain reduction with 244 of 299 (81.6%) individuals reporting good, very good, or excellent on the global assessment of pain. Only about one in eleven (8.8%) non-surgical patients and approximately one in five surgical patients (18.0%) rated suzetrigine’s overall effectiveness at the end of 2 weeks as “poor” or “fair” (38).

Some caveats regarding the (38) trial are also noteworthy. Similar to the previous trials (36, 37), suzetrigine was not assessed as a monotherapy, with almost three-quarters (73.0%) of participants using ibuprofen or acetaminophen as part of multimodal treatment (38). The maximum allowable acetaminophen dose was 2,600 mg and ibuprofen was 1,600 mg in a 24 h (SAP, 2023)^3^. Three pre-specified outcomes from the SAP (Vertex, 2023)^4^ were absent from the published paper and supplementary materials: mean daily rescue medication dose (acetaminophen and ibuprofen reported separately), attribution of pain resolution to suzetrigine versus rescue medication, and suicidality assessed via the Columbia Suicide Severity Rating Scale. Inclusion of these details would have facilitated a more thorough assessment of the trial results.

Beyond the lumbosacral radiculopathy trial’s failure to separate from placebo, the absence of a peer-reviewed publication warrants attention. Best practices in clinical research call for summary results to be posted in clinical trial registries within 12 months of study completion and published in a peer-reviewed journal within 24 months (46). This standard is particularly important for a first-in-class analgesic targeting one of the most common reasons patients seek primary care. Although Vertex has consistently registered trial methods at ClinicalTrials.gov, including the Phase II acute pain (NCT05160636), diabetic peripheral neuropathy (NCT05660538), and lumbosacral radiculopathy (NCT06176196) trials, registration satisfies a regulatory minimum but is not a substitute for peer-reviewed publication. Major publishers routinely publish null findings, and registered reports, in which methods are peer-reviewed prior to data collection, are an established mechanism for ensuring the scientific record does not reflect selective reporting of results. As of this writing, the lumbosacral radiculopathy null result has been available only as a corporate press release for 17 months since December 2024 (58). For a drug now approved and actively prescribed for acute pain, this gap in the published literature represents a meaningful deficit in the evidence base available to clinicians.

## 7 Discussion

There are 80.2 million people in the US that experience pain necessitating prescription medication (47). Suzetrigine represents a meaningful scientific advance because it is the first isoform-selective VGSC inhibitor to demonstrate Phase III success in human pain, validating a long-standing hypothesis that peripheral, molecularly targeted analgesia can achieve pain relief with minimal engagement of CNS pain circuitry. By selectively stabilizing Nav1.8 in its closed state through allosteric binding to the VSD2 domain, suzetrigine provides a potentially mechanistically precise way to suppress nociceptor excitability while avoiding the broad, non-selective VGSC blockade that has historically limited other pharmacological agents. This advance potentially marks a turning point in mechanism-based drug development: It confirms that targeting a single nociceptor-specific VGSC isoform, rather than modulating multiple CNS pathways, can produce analgesia with a substantially improved safety profile (19). As the first clinical validation of Nav1.8 as a druggable pain target, suzetrigine establishes a new therapeutic framework for peripherally targeted analgesics and is generally encouraging for the development of future isoform-selective VSGC modulators (48).

In the realm of analgesia and acute postoperative pain contexts, suzetrigine is emerging as a potentially meaningful contributor to pain reduction. Clinical trials showed that pain reduction was found to be comparable, but not superior, to opioid comparators, highlighting that suzetrigine should be viewed primarily as an additional pain management tool rather than a replacement for opioids (36–38). Use of suzetrigine alongside other analgesics, although promising, has yet to be investigated directly. The adverse effects of suzetrigine were relatively mild including headaches, dizziness, nausea, and constipation, and serious adverse events were rare in the clinical trials and assumed to be unrelated to suzetrigine treatment, but further studies are also needed to more fully understand the nature of these adverse reactions considering the assumed peripherally-acting mechanistic basis for suzetrigine. Importantly, neither respiratory depression nor evidence of drug misuse, common challenges associated with opioid-based treatments, were reported in any of the clinical trials (19, 36–38). Taken together, although more animal model and clinical studies are needed, the outlook for suzetrigine is encouraging.

Despite promising results to date, we and others have identified important limitations to the studies in which suzetrigine has been investigated and gaps which need to be addressed with additional research (2, 40, 41, 49). From a mechanistic standpoint, Nav1.8 is expressed in intracardiac neurons where it contributes to AP frequency and overall cardiac electrical activity (25, 50). This raises the risk that suzetrigine could exhibit deleterious impacts on cardiac function in patients beyond the intended analgesic effects. Additional studies in model systems and patients are needed to clarify the impact that suzetrigine has on the excitability of cardiomyocytes and intracardiac neurons as well as overall cardiac function. Given the abundant use of rescue medication in Phase II/III trials (36–38), an additional preclinical priority would be to complete an extended (≥ 2 weeks) head-to-head comparison of suzetrigine, as a monotherapy, with a single NSAID at a high dose, also as a monotherapy, and subsequently completing trials of both pain and adverse effects from multiple organ systems in humans including headache, constipation, nausea, and dizziness (51). Many over the counter NSAIDs (e.g., diclofenac, ketorlak, ibuprofen) already have a well-established profile in adults from randomized controlled trials for their opioid sparing effects as well as acute and intermediate term (weeks) adverse effects (42).

Conclusions regarding the long-term efficacy of suzetrigine are constrained by trial design. While the pivotal Phase III surgical trials assessed the primary efficacy endpoint over 48 h (SPID48), total treatment duration across all Phase III studies was limited to 14 days (36, 38). Whether selective Nav1.8 inhibition confers meaningful benefit in persistent or chronic pain states remains an open question, as current clinical evidence is derived almost exclusively from acute postoperative and short-term non-surgical contexts (39, 40). Data on more diverse patient populations including elderly patients and people with several comorbid diseases, who frequently have altered pharmacokinetics or are more vulnerable to negative pharmacological effects, are still scarce because current research have also used relatively controlled (i.e., homogeneous and single pain types) patient populations (36, 52).

Suzetrigine is not only an important development for the goal of generating efficacious non-opioid analgesics, but it also highlights the potential for isoform-specific VGSC targeting for the treatment of other channelopathy-related diseases (8, 48). Recent advances in the structural biology of voltage-gated sodium channels and artificial intelligence, have enabled researchers to visualize VGSC structures with greater precision, identify isoform-specific binding pockets, and develop more selective inhibitors (48). Driven by highly specific expression across the body and over developmental time, each VGSC isoform is associated with unique physiological function, and when aberrantly functioning, specific pathophysiologies including epilepsy, cardiac arrythmia, sensory and movement dysfunction, pain, and others. Beyond the development of Nav1.8-specific blockers, specific inhibitors of Nav1.2 (53), Nav1.3 (54), Nav1.6 (16), and Nav1.7 (55–57) are at various stages of preclinical and clinical investigation. Suzetrigine offers a possible glimpse of the clinical future for the use of these targeted therapeutic agents and serves as a model for future ion channel drug development. Yet suzetrigine’s limitations should also be taken in to account. This includes considerable debate whether the benefits of suzetrigine are clinically meaningful (41). The existing clinical trials should be viewed with caution due to a pattern of deviating from the registered methods and selective reporting of dependent measures.

Suzetrigine represents a genuine pharmacological milestone. It is the first non-opioid to be approved for acute pain in the US in over two-decades (24), and it is the first proof-of-concept that isoform-specific targeting of voltage-gated sodium channels can translate to help patients. The broader promise of this approach, in which distinct channel-related pathological states drive the selection of highly specific pharmacological agents, positions suzetrigine as the first entry in what will hopefully become a new class of precision therapeutics. However, the clinical evidence base supporting the current approval and use of suzetrigine has multiple meaningful limitations underscoring the need for future rigorous studies before the promise of isoform-specific VGSC pharmacotherapy can be realized.

## 8 Conclusion

Suzetrigine is an important milestone in the advancement of non-opioid analgesics, presenting a unique and decisive approach to acute pain management through the selective inhibition of the peripherally expressed Nav1.8. Phase II and III clinical trials have demonstrated some degree of effectiveness with effect sizes often that are small or, at best, moderate of this mechanism-based strategy, confirming that targeting nociceptor activity at its source can yield meaningful analgesia without engaging central opioid pathways. By validating Nav1.8 as a druggable and clinically impactful target that produces pain relief often within weeks but not hours, suzetrigine establishes a new framework for precision analgesia, one that prioritizes isoform selectivity, a peripherally-restricted molecular target, and improved safety profiles over traditional agents like opioids and NSAIDs. Although its long-term durability, applicability to chronic pain states, and performance in diverse patient populations remain areas for continued investigation, the current evidence positions suzetrigine as a foundational step toward safer and more targeted pain therapeutics. As research expands, the success of suzetrigine will hopefully catalyze the development of additional isoform-selective sodium-channel modulators, marking a broader shift toward mechanism-driven innovation in pain management.

## Footnotes

1
https://www.accessdata.fda.gov/drugsatfda_docs/label/2025/219209s000lbl.pdf

2
https://cdn.clinicaltrials.gov/large-docs/36/NCT04977336/SAP_001.pdf

3
https://icer.org/assessment/acute-pain-2025/

4
https://cdn.clinicaltrials.gov/large-docs/34/NCT05661734/SAP_001.pdf
