# Supplementary material for: From ion channel biology to clinical practice: suzetrigine as a precision analgesic
Source: Front Med (Lausanne). 2026 Jul 29;13:1897421. doi: 10.3389/fmed.2026.1897421 (PMC13463154; doi:10.3389/fmed.2026.1897421)
Supplement: Supplementary file 1 [file Supplementary_file_1.docx]

**Supplementary Materials**

**Supplemental Figure 1.** No significant effect of hydrocodone (HC, 5 mg) / acetaminophen (325 mg) on pain ratings (*±* SEM) at 48-hours in the Jones et al. 2023 Phase II abdominoplasty (*t*(117) = .894, p = .373 ) or bunionectomy (*t*(117) = .894, *p* = .373) trials.

**Supplemental Table 1.** Non-parametric analysis (chi-square, two-tailed) of the time-course of the percent of patients with Numeric Pain Rating scale 11-point (0 to 10) pain rating of 30, 50 or 70% reductions in the Phase II randomized clinical trials of VX-548 (suzetrigine) by Jones et al. 2023. Out of 21 statistics with *p* < .05 versus placebo, one was statically significant. APAP: acetaminophen (325 mg, every 6 hours); Hyd: hydrocodone (5 mg, every 6 hours), NA: Not Applicable.

_________________________________________________________________________________________________________________

Hyd /	Low	Middle	High

*Abdominoplasty*	Placebo (%)	APAP	(%)	*p*	VX-548	*p*	VX-548	*p*	VX-548	*p*

30%	48.1%	53.9%	.4658	NA	NA	59.5%	.1600	60.5%	.1215

50%	33.8%	42.1%	.2878	NA	NA	43.2%	.2313	44.7%	.1646

70%	14.3%	23.7%	.1381	NA	NA	18.9%	.4438	25.0%	.0951

*Bunionectomy*

30%	67.8%	68.3%	.9499	75.8%	.4212	62.9%	.5720	83.3%	.0484

50%	61.0%	61.7%	.9420	72.7%	.2580	56.5%	.6102	66.7%	.5212

70%	40.7%	50.0%	.3071	51.5%	.3158	38.7%	.8249	51.7%	.2293

_________________________________________________________________________________________________________________
